# Factors associated with rates of tobacco treatment delivery by General Practitioners in Greece: Missed opportunities for prevention?

**DOI:** 10.18332/tid/90822

**Published:** 2018-05-21

**Authors:** Sophia Papadakis, Charis Girvalaki, Constantine Vardavas, Andrew L. Pipe, Adam Cole, Ioanna Tsiligianni, Eleni Petridou, Christos Lionis

**Affiliations:** 1 Clinic of Social and Family Medicine, Department of Medicine, University of Crete, Heraklion, Greece; 2 Division of Prevention and Rehabilitation, University of Ottawa Heart Institute, Ottawa, Canada; 3 Faculty of Medicine, University of Ottawa, Ottawa, Canada; 4 School of Public Health and Health Systems, University of Waterloo, Waterloo, Canada; 5 Department of Hygiene, Epidemiology and Medical Statistics, Medical School, National and Kapodistrian University of Athens, Athens, Greece

**Keywords:** tobacco treatment delivery, factors, primary health care, Crete

## Abstract

**INTRODUCTION:**

This study investigates the clinic-, provider- and patient-level factors associated with delivery of 4 (Ask, Advise, Assist, Arrange) elements of the 5As approach to smoking cessation in general practice in Greece.

**METHODS:**

We conducted a secondary analysis of data derived from a quasi-experimental study (The TiTAN Crete study) among general practitioners (GPs) in Crete, Greece in 2015–2016. Twenty-four GPs and a cross-sectional sample of 1301 smokers from their practices were surveyed. This paper reports on the results of the multi-level modelling conducted to examine predictors of 4As delivery.

**RESULTS:**

Our analysis found clinic characteristics, including the presence of an electronic medical record, being located in a rural setting, and being in private practice were significantly associated with increased rates of tobacco treatment delivery. Female GPs were more likely than males to arrange follow-up (AOR 3.38, 95%CI 1.11, 10.35). Our analysis found a variety of patient-level factors were positively associated with tobacco treatment delivery, including: longer smoking history; presence of a smoking related illness; readiness to quit smoking; and symptoms or a diagnosis of anxiety, depression or other mental health illness. Other patient-level factors were negatively associated with tobacco treatment delivery, including level of education and reason for visit. Patients seen in clinic for episodic care were less likely to be ‘asked’ (AOR 0.22, 95%CI 0.12, 0.39), ‘advised’ (AOR 0.22, 95%CI 0.13, 0.38), and receive ‘assistance’ (AOR 0.36, 95%CI 0.19, 0.66) compared to patients seen in clinic for a medical examination.

**CONCLUSIONS:**

Providers are significantly more frequently delivering tobacco treatment to a sub-group of high-risk patients compared to other tobacco users in their clinical practice. This results in missed opportunities for early intervention and disease prevention.

## INTRODUCTION

Tobacco use remains the leading cause of preventable disease and death in Europe and worldwide^1^. The World Health Organization (WHO) and the European Tobacco Treatment Guidelines have recognized tobacco use as a disease and recommend tobacco treatment as a priority for the prevention and control of chronic diseases in primary care practice^2,3^.

According to the latest Special Eurobarometer report on the attitudes of Europeans towards tobacco and electronic cigarettes, Greece has the highest prevalence of smoking among European member states: one-third of adults are smokers (37.0%) while one-fifth of adult smokers (20.0%) have never tried to quit smoking^4^. Of those who tried, or that have quit smoking, the vast majority did so unassisted^4^.

General practitioners (GPs) are ideally positioned to deliver tobacco treatment interventions^2^ for several reasons: GPs interact with a large part of the population regularly^5^; tobacco treatment delivery may be more acceptable, given a GP’s role to prevent disease and promote healthy lifestyle^6^; and GPs have established trusted interpersonal relationships with their patients^7,8^. As a result, clinical practice guidelines for tobacco treatment delivery recommend that GPs use the following ‘5As’ approach for addressing tobacco use in clinical practice: *Ask* about tobacco use at every visit; *Advise* smokers to quit; *Assess* smokers’ readiness to quit; *Assist* smokers to quit using a combination of behavioral counseling and pharmacotherapy; and *Arrange* follow-up visits to review progress, address any problems and anticipate future challenges in order to prevent smokers from relapsing^2,3,9^.

Despite clear guidelines, many GPs find it difficult to integrate tobacco treatment delivery into their daily clinical practice^10,11^. Previous evaluations report significant variation in the rates at which the ‘5As’ are delivered by GPs to patients, even among GPs within the same clinic^12,13^. Multi-component interventions, are interventions that combine two or more intervention strategies^14,15^. There is good evidence from meta-analyses to show that multi-component interventions that include training and other provider- and patient-level supports increase rates of 5As delivery in primary care practice settings^7,14,15^. The effectiveness of multi-component interventions is hypothesized to be related to the fact that they can address the multiple barriers to tobacco treatment delivery in the primary care setting^14^. However, variations in the rates at which providers intervene with patients who smoke continue to be documented even following exposure to multi-component interventions^12^. We hypothesize that increased knowledge of the clinic-, provider-, and patient-level factors that are associated with delivery of the ‘5As’ could aid the identification of sub-populations that might benefit from interventions to increase tobacco treatment delivery.

The purpose of this study was to investigate the clinic-, provider- and patient-level determinants of tobacco treatment delivery for 4 (Ask, Advise, Assist, Arrange) of the 5As in general practices in Crete, Greece. The present study did not examine the ‘Assess’ strategy as part of the intervention, as it was not of specific interest to investigators.

## METHODS

### Design and setting

We conducted a secondary analysis of data generated from a quasi-experimental study (TiTAN Crete) to examine predictors of 4As delivery using three level (clinic, provider, patient) multi-level modelling. The study took place in general practices on the island of Crete in Greece. Data were collected from the practices of providers and from a sample of their patients who smoke.

### The TiTAN Crete Study

The TiTAN Crete project examined the impact of a multi-component intervention to increase rates of ‘4As’ tobacco treatment delivery using a quasi-experimental non-randomized controlled design. The study was approved by the University Hospital of Heraklion Ethics Board and registered on ISRCTN #10306198. The full study protocol^16^ and main results^12^ have previously been published.

As part of the TiTAN Crete study, all GPs (n=26) in the geographically defined intervention and control regions of Crete were invited to participate. Twenty-four GPs agreed to participate in the study, provided informed consent and completed a baseline survey. GPs (n=14) in the city of Heraklion were exposed to the intervention programme and acted as the intervention group, while those in the city of Rethymnon (n=10) were not exposed to the intervention programme and acted as the control group. Independent, cross-sectional samples of eligible patients were recruited from practices in the intervention group before (May–September 2015) and after (March–May 2016) exposure to the intervention-training programme (September 2015). Similarly, a cross-sectional sample of eligible patients from practices in the control group was recruited, but only at one time point (between December – May 2016), as it was assumed that no changes in outcomes would occur during the short time period of the study. Patients were screened for eligibility in the waiting rooms of all participating GP offices. Eligibility criteria included being: 18 years of age or older; current tobacco users (≥1 cigarette per day); seen in clinic for a non-urgent medical visit; and able to read/understand Greek. Eligible patients who agreed to participate in the study provided informed consent and completed the study survey at the end of their clinic appointment. This methodology was repeated four to six months following implementation of the intervention programme, in the intervention group only.

### The TiTAN Crete Intervention

The TiTAN Crete intervention programme was based on the Ottawa Model for Smoking Cessation (OMSC, University of Ottawa Heart Institute), an evidence-based intervention tested in primary care practices in Canada. The OMSC intervention was adapted to reflect the local language, cultural norms related to tobacco use, the health system, and GPs clinical practice routines in Greece^13-16^. The intervention programme, which has been previously described, consisted of a 1-day core tobacco dependence treatment training programme, two booster training sessions lasting 2.5 hours each delivered at 2 and 4 months after the initial training, and the dissemination of GP and patient clinical resources to support the integration of evidence-based tobacco treatment into daily clinical routines^16^. The resources included: a patient tobacco-use survey, a provider smoking cessation consult form, provider quick reference sheets, patient quit-plan booklets, and posters. The training materials and resources are available at www.titan.uoc.gr. The control group was not exposed to any intervention programme.

### Measures

#### Outcomes: GP performance in ‘4As’ delivery

Performance of ‘4As’ delivery (‘Ask’, ‘Advise’, ‘Assist’, ‘Arrange’) was assessed using a patient exit survey. The survey asked participants to respond either ‘yes’, ‘no’ or ‘don’t know’ regarding whether on the same day of their visit to the clinic (‘index visit’) the GP asked them if they smoke (‘Ask’); advised them to quit (‘Advise’); provided help material; and arranged follow-up support (‘Arrange’), including scheduling a follow-up for assistance to quit (‘Assist’), with respondents prompted with examples of assistance (i.e. set a quit date, provided pharmacotherapy, provided counselling, provided self-visit at the clinic or referral to a specialized hospital-based quit smoking clinic.

#### Predictor variables

Clinic-, provider- (i.e., GP) and patient-level variables thought to be associated with rates of tobacco treatment delivery were assessed. Clinic-level variables assessed included: exposure to the TiTAN Crete training intervention (yes/no), the geographic location of clinic (urban/rural/suburban), reimbursement method (fee for service or salaried) and type of record system. Provider-level variables assessed included: age, gender, number of years practicing medicine, previous cessation training, and personal tobacco use. Patient-level variables assessed included: age, gender, nationality, formal education, and current or past smoking-related illness (e.g. heart disease, stroke, chronic obstructive pulmonary disease, and cancer). The Greek validation of the 4-item Patient Health Questionnaire (PHQ-4) was used to screen for anxiety and depression^17,18^. Participants were also asked to report if they had been diagnosed with anxiety, depression or mental health illness in the past. Smoking related variables included two variables from the Heaviness of Smoking Index (HSI)^19,20^, including time to first cigarettes in the morning and number of cigarettes smoked per day (CPD). Number of years of tobacco use was documented. Patient self-efficacy (‘On a scale of 1 to 10 how confident are you that you would be able to quit smoking at this time?’) and readiness to quit smoking (‘Which of the following best describes your feelings about smoking right now?’) were also assessed as well as the purpose of the clinic visit.

#### Secondary analysis & multi-level modelling procedures

Descriptive statistics summarized characteristics of the sample at the clinic-, provider- and patient-levels. To examine clinic-, provider-, and patient-level factors associated with each outcome (i.e. performance of ‘4As’), separate multi-level logistic regression analyses were performed. We included patients from the ‘after’ cross-sectional sample in the intervention group and the cross-sectional sample in the control group for comparison. Intervention group (control=0, intervention=1) was included as a variable in the model to account for the potential effect of the TiTAN intervention. The model building followed a step-wise approach whereby significant variables (p<0.1) from each level (clinic, provider, and patient) were included in each step. Only those variables significant at p<0.05 were kept in the final model. Results were reported as adjusted odds ratios (AOR) and 95% confidence intervals (95%CI). We used a cut off score of ≥3 on the PHQ as a positive screen for anxiety or depression^17,21^. For the multilevel analysis we created a combined variable (1 = a positive screen for anxiety or depression or a patient self-reported diagnosis of anxiety, depression or other mental health illness being, and 0=all other patients).

## RESULTS

A sample of 1301 patients who smoked was recruited from control and intervention clinics and was included in the analysis. The recruitment rate was 98.8% of eligible patients screened. Characteristics of the clinics, providers, and the patients sampled are presented in Table 1.

**Table 1:** Characteristics of clinics, providers and patients sampled

*Parameter*	*Response*	*Value*
**Clinic-level variables**		
Geographic location	Urban	8.3%
	Suburban	20.8%
	Rural	70.8%
Type of record system	Electronic	36.4%
	Manual	27.3%
	Both	36.4%
Reimbursement method	Fee for service (private)	12.5%
	Salaried (public)	87.5%
**Provider-level variables**		
Gender	Female	54.2%
	Male	45.8%
Years of practicing medicine	Mean (SD)	13.8 (4.9)
Age	30–39 years	20.0%
	40–49 years	70.0%
	50–59 years	10.0%
Previous smoking cessation training	No	70.0%
	Yes	30.0%
Smoking status	Smoker	25.0%
	Ex-smoker	33.3%
	Non-smoker	41.7%
**Patient-level variables**		
Age	Mean years (SD)	48.2 (14.0)
Gender	Female	41.6%
	Male	58.4%
Education	Grade school	21.2%
	Junior high school	20.9%
	High school	30.6%
	College/University	27.3%
Nationality	Greek	97.9%
	Other	2.1%
Smoking-related illness^a^		18.4%
Anxiety, depression or other mental health illness		13.2%
Depressive symptoms^b^	PHQ score >3	8.1%
Anxiety symptoms^c^	PHQ score >3	20.1%
Purpose of visit	Medical examination	44.9%
	Prescription	38.6%
	Other	16.5%
Cigarettes/day	<15	21.1%
	15–20	48.3%
	>20	30.6%
Time to first cigarette in am	>30 minutes	30.5%
	<30 minutes	69.5%
Years of tobacco use	0–2 years	1.5%
	3–9 years	7.1%
	10–19 years	22.2%
	20+ years	69.2%
Readiness to quit^d^	Next 30 days	19.9%
	Next 6 months	41.3%
	Not ready to quit	38.8%
Self-efficacy with quitting^e^	Low (<7/10)	87.0%
	High (>7/10)	13.0%

a Do you have... heart disease, stroke, heart failure/cancer/chronic obstructive pulmonary disease (COPD)? (1=yes, 0=no).

b Positive screen (score of 3 or more) on Patient Health Questionnaire (PHQ)-4 for Depression.

c Positive screen (score of 3 or more) on Patient Health Questionnaire (PHQ)-4 for Anxiety.

d Which of the following best describes your feelings about smoking right now? (responses: 1=ready to quit in next 30 days, 0= ready to quit in next 6 months or not ready to quit).

e On a scale of 1 to 10 how confident are you that you would be able to quit smoking at this time? (1=not at all confident, 10=extremely confident).

### Effects of the TiTAN Intervention

The analysis documented that following the intervention; GPs in the intervention group were significantly more likely to deliver each of the ‘4As’ during their daily clinical practice compared to those in the control group (Table 2).

**Table 2:** Final model examining clinic-, general practitioner-, and patient-level characteristics associated with rates of 4As tobacco treatment delivery

*Adjusted Odds Ratio (95%CI)*^a^				
*Parameter*	*ASK*	*ADVISE*	*ASSIST*	*ARRANGE*
**Intervention characteristics**				
**Training intervention**				
Not exposed	1.00	1.00	1.00	1.00
Exposed	3.11 (1.11, 8.68)*	4.60 (2.04, 10.36)***	68.26 (29.61, 157.37)***	22.65 (5.35, 95.78)***
**Clinic-level variables**				
**Geographic location**				
Rural	-	-	1.00	1.00
Suburban			0.30 (0.14, 0.67)**	0.18 (0.05, 0.67)*
Urban			0.23 (0.04, 1.49)	2.28 (0.63, 9.86)
**Type of record system**				
Manual	1.00	1.00	-	-
Electronic	5.03 (1.25, 20.18)*	4.59 (1.53, 13.76)**		
**Reimbursement method**				
Fee for service (private)	-	-	1.00	
Salaried (public)			0.19 (0.04, 0.85)*	
**Provider-level variables**				
**Gender**				
Male	-	-	-	1.00
Female				3.38 (1.11, 10.35)*
**Patient-level variables**				
**Education**				
Grade school	1.00	1.00	-	-
Junior high school	0.45 (0.24, 0.86)*	0.49 (0.26, 0.92)*		
High school	0.68 (0.37, 1.27)	0.60 (0.33, 1.10)		
College/University	0.86 (0.43, 1.71)	0.84 (0.43, 1.62)		
**Smoking-related illness**				
No	-	-	1.00	1.00
Yes			2.75 (1.55, 4.88)***	2.88 (1.47, 5.65)**
**Symptoms or a diagnosis Anxiety, depression, or other mental illness^b^**				
No	-	-	1.00	1.00
Yes			2.47 (1.28, 4.78)**	2.18 (1.08, 4.41)*
**Years of tobacco use**				
0–2 years	1.00	1.00	-	-
3–9 years	5.55 (1.14, 27.09)*	3.19 (0.71, 14.24)		
10–19 years	5.04 (1.29, 19.79)*	3.72 (1.00, 13.83)*		
20+ years	6.39 (1.70, 24.01)**	4.79 (1.34, 17.08)*		
**Readiness to quit^c^**				
Not ready in the next 30 days	1.00	1.00	1.00	1.00
Ready in the next 30 days	1.85 (1.01, 3.39)*	2.08 (1.16, 3.74)*		
**Purpose of visit**				
Medical examination	1.00	1.00	1.00	1.00
Prescription	0.78 (0.48, 1.25)	0.71 (0.45, 1.12)	0.59 (0.36, 0.94)*	
Other/Missing	0.22 (0.12, 0.39)***	0.22 (0.13, 0.38)***	0.36 (0.19, 0.66)**	
**Random Variance**				
General practitioner	0.984 (0.449)	0.557 (0.282)	0.251 (0.180)	0.638 (0.380)

Final Model Ask: 8 clinics, 24 general practitioners; 1= Asked about smoking (n=529), 0= Not asked about smoking (n=173).

Final Model Advise (overall): 8 clinics, 24 general practitioners; 1= Advised to quit smoking (n=510), 0= Not advised to quit smoking (n=192).

Final Model Assist (overall): 8 clinics, 24 general practitioners; 1= Assisted with quitting (n=324), 0= Not assisted with quitting (n=449).

Final Model Arrange: 8 clinics, 24 general practitioners; 1= Arranged follow-up (n=73), 0= Did not arrange follow-up (n=702).

p-values calculated based on Wald Tests.

* p<0.05;

** p<0.01;

*** p<0.001.

a Models adjusted for general practitioner-level clustering effects; CI = confidence interval.

b Self-reported positive screen (>3) on PHQ-4 or diagnosis of anxiety, depression or other mental health illness.

c Which of the following best describes your feelings about smoking right now? (responses: 1=ready to quit in next 30 days, 0= ready to quit in next 6 months or not ready to quit).

### Predictors of ‘4As’ Delivery

#### Clinic-level factors

GPs working in clinics with an electronic medical record were more likely to ‘ask’ (AOR 5.03, 95%CI 1.25, 20.18; p<0.05) and ‘advise’ (AOR 4.59 95%CI 1.53, 13.76; p<0.01) patients to quit smoking relative to a manual record system (Table 2). Rates of ‘assist’ and ‘arrange’ were significantly lower among GPs in suburban practices compared to rural settings (AOR 0.30, 95%CI 0.14, 0.67; p<0.01, and AOR 0.18, 95%CI 0.05, 0.67; p<0.05, respectively). Being a GP from a salaried Health Care Centre was significantly associated with decreased rates of ‘assist’ compared to those in private practice (AOR 0.19, 95%CI 0.04, 0.85; p<0.05).

#### Provider-level factors

Female providers were more likely to ‘arrange’ follow-up support relative to male providers (AOR 3.38, 95%CI 1.11, 10.35; p<005), although the confidence intervals are quite wide (Table 2). None of the other GP-level variables examined were found to be significant in predicting ‘4As’ delivery.

#### Patient-level factors

Patients with a junior high school education or less were less likely to be ‘asked’ (AOR 0.45, 95%CI 0.24, 0.86; p<0.05) and ‘advised’ (AOR 0.49, 95%CI 0.26, 0.92; p<0.05) about tobacco use than those with a grade school education (Table 2). Having a smoking-related illness was positively associated with increased frequency of delivery of ‘assist’ (AOR 2.75, 95%CI 1.55, 4.88; p<0.001) and ‘arrange’ (AOR 2.88, 95%CI 1.47, 5.65; p<0.01). A positive screen for anxiety or depression or self-reported diagnosis of anxiety, depression, or mental health illness was also associated with higher rates of ‘assist’ (AOR 2.47, 95%CI 1.28, 4.78; p<0.01) and ‘arrange’ (AOR 2.18, 95%CI 1.08, 4.41; p<0.05). Individuals who smoked for more than 2 years were more likely to be ‘asked’ and ‘advised’ relative to those who smoked for less than 2 years. Individuals reporting a readiness to quit in the next 30 days were more likely to be ‘asked’ (AOR 1.85, 95%CI 1.01, 3.39; p<0.05) and ‘advised’ (AOR 2.08, 95%CI 1.16, 3.74; p<0.05) to quit smoking relative to those who did not report being ready to quit smoking in the next 30 days. Patients seen by the GP for prescription were less likely to be ‘assisted’ (AOR 0.59, 95%CI 0.36, 0.94; p<0.05) relative to an appointment for a medical examination.

## DISCUSSION

Several clinic- and patient-level factors were associated with increased likelihood of receiving ‘4As’ tobacco treatment. Specifically, patients with a smoking related illness, mental health diagnoses and a greater number of years smoking were more likely to receive cessation treatment. These trends were reduced but not eliminated by exposure to the training-based intervention. Similar trends reflecting this selection bias in the delivery of tobacco treatment have recently been reported among GPs sampled in Canada^21,22^. Our findings are consistent with previous research which found that ‘advice’ to quit smoking is delivered more frequently in primary care to individuals with a smoking-related illness^22,23^, a smoking history of 20 or more years, and higher levels of nicotine dependence^24^. While this group of ‘high risk’ patients are important targets for intervention, and may be most open to intervention, best practice guidelines call for ‘advise’ and ‘assistance’ with quitting to be delivered to all patients, at all visits^2,3,9^.

Patients were less likely to receive intervention when seen in clinic for episodic visits or visits for a prescription refill compared to appointments for medical examinations. This may be a function of the opportunistic discussion or time typically afforded to medical examination versus appointment for medication refills. Given that brief advice can be delivered in a short period of time this may indicate the existence of other barriers (e.g. attitudinal, skill), which may be the focus of future interventions. Additionally, as has been reported by others, female GPs were more likely to arrange follow-up support with patients^21,25^. There was a positive association with having an electronic medical record system and the odds of ‘ask’ and ‘advise’, which attests to the possible benefit of including reminders in electronic record systems as has been reported by others^26^.

### Implications for future practice and research

Our study has documented the missed opportunities for early intervention and prevention in addressing tobacco use with all patients who smoke in general practices sampled in Crete, Greece. The patterns observed may be associated with provider beliefs about the importance of cessation once patients are at increased ‘risk’ relative to ‘healthy’ patients. Likewise, it is also possible that provider beliefs about patient readiness to quit and/or openness to listen to advice and intervention about smoking cessation may play a role in the observed trends. Perhaps critical to this discussion and future interventions is an understanding that all patients who smoke are at enormous risk of disease, disability, and death^1^. Gold standard evidence has shown that one in every two smokers will die of tobacco-related illness^27^. Moreover, quitting as early as possible, in particular before the age of 40 is the only method for reducing the devastating effects of smoking^27^. Our study’s findings and those of previous researchers suggest clinicians have a tendency to wait until a patient is diagnosed with a smoking-related illness or begin to see measurable consequences of tobacco use before intervening with cessation assistance^22,23^. Interestingly, the predictors identified differed for each of the 4As suggesting each is a distinct step with its own set of determinants and as such may require different intervention strategies in order to increase treatment rates. A finding that has been previously reported in the literature^14,21,22,28^.

Investigations, such as our own, which examine the patterns associated with tobacco treatment delivery in primary care can assist with designing future training interventions for GPs with the goal of ensuring all patients who smoke receive regular advice, motivational interventions, and evidence-based cessation treatments for smoking cessation. Furthermore, the availability of educational and motivational interventions to assist patients who might still be disease- or symptom-free to comprehend the significant risk imposed by their tobacco addiction may assist with increasing treatment rates.

At a time in which Greece and other European countries are reforming their primary health care systems, supporting the primary care practitioners’ role as ‘gate-keepers’ of patient and community health and ensuring the early detection, prevention and management of chronic diseases is critical. Central to this role is the responsibility to address tobacco use with all patients who smoke within a primary care clinical practice.

### Strengths and limitations

The multi-level analysis used in the present study allowed us to examine multiple factors associated with rates of tobacco treatment delivery in primary care practice. Moreover, we have examined factors at three levels: clinic, provider, and patient. The present study had very high rates of participation from both GPs and patients, and as such is highly representative of patients and providers in the primary care practices sampled. The study included representation from GPs in rural, semi-urban, and remote areas, which permits an assessment of the generalizability of the findings to different clinical practices. The inclusion of rural and remote settings is infrequently seen in the smoking cessation literature examining primary care. Our findings are derived from primary care practices on the island of Crete in Greece and as such may not be generalizable to other settings or countries. We examined specific characteristics hypothesized to be of relevance to the delivery of tobacco treatment, which were collected as part of the TiTAN Crete study. Not all variance observed could be explained by the factors examined, particularly at the provider level. It is possible that a more in-depth examination of predictors could further explain the variance in rates of tobacco treatment and should be the focus of future research. Finally, the present study did not examine the ‘Assess’ component of the ‘5As’ model, in order to reduce participant burden, as this variable was not of specific interest to study investigators. It would be important to note measurement of 4 of the 5As does not affect the validity of the study instrument or pose any significant limitations to the study findings in our opinion.

## CONCLUSIONS

Our data suggest providers may be significantly more frequently delivering tobacco treatments to a sub-group of high-risk patients in comparison to other tobacco users in their practices. Given the importance of intervening with all patients who smoke, efforts should focus on strategies to reach a larger proportion of the patient population that smokes in order to optimize opportunities for early intervention and prevention of disease.
